# Limited effects on patient outcomes of conjoint tendon release in anterolateral muscle-sparing total hip arthroplasty

**DOI:** 10.1186/s13018-021-02644-7

**Published:** 2021-08-10

**Authors:** Hidetatsu Tanaka, Norikazu Yamada, Hiroaki Kurishima, Yu Mori, Takashi Sakamoto, Masamizu Oyama, Toshimi Aizawa

**Affiliations:** 1 Department of Orthopaedic Surgery, Japanse Redcross Sendai Hospital, 43-3, 2 cho-me, yagiyama hon-cho, taihaku-ku, Sendai, 982-8501 Japan; 2 Department of Orthopaedic Surgery, Tohoku University Graduate School of Medicine, 1-1 Seiryo-machi, Aoba-ku, Sendai, 980-8574 Japan

**Keywords:** Total hip arthroplasty, conjoined external rotators tendon, patient outcome

## Abstract

**Background:**

The anterolateral muscle-sparing total hip arthroplasty (THA) in the supine position is advantageous owing to the very low-dislocation rate and excellent leg length discrepancy control. However, femur exposure is challenging. Although the conjoined external rotators tendon (CERT) release is effective in improving femoral access, the effects on clinical outcomes remain unclear. The purpose of this study was to evaluate the clinical and radiographic results of CERT release in the anterolateral muscle-sparing THA approach.

**Methods:**

The study was performed as a retrospective cohort study and included 85 hips in 85 patients who underwent primary anterolateral THA. Clinical and radiographic outcomes were investigated 6 months and 1 year after THA (CERT-preserved and non-released patients). The Japanese Orthopaedic Association (JOA) hip score, JOA Hip-disease Evaluation Questionnaire (JHEQ), forgotten joint score (FJS), and the 36 short-form questionnaires (SF-36 mental and physical) were evaluated. The leg length discrepancy, cup inclination and stem orientation were evaluated with radiographs.

**Results:**

Among all the included hips, 37 patients (43.5%) retained the CERT, and 48 patients (56.5%) included the released CERT. There were no significant differences in the JOA hip scores, JHEQ, FJF-12 and SF-36 between the released and non-released groups. There were significant differences in sagittal stem alignments between groups.

**Conclusion:**

The CERT release in anterolateral muscle-sparing THA has a limited effect on post-operative clinical outcomes. The CERT release improved the femur exposure and is more invasive than the preserved CERT. We infer that the CERT should be maintained in patients with a wide range of motions, and release the CERT in inadequate femur canal preparation cases.

## Introduction

Total hip arthroplasty (THA) provides pain relief and restoration of functionality in osteoarthritic hips and leads to the improvement of the patients’ quality of life [1]. Recent studies have demonstrated that THA with the minimally invasive techniques is effective in reducing soft tissue damage, peri-operative pain, blood loss, and achieves faster recovery times and shorter hospitalization periods [2–5]. The anterior-based muscle-sparing (ABMS) THA is less popular than the direct anterior approach (DAA) applied to the hip, clinically effective, safe, very low-dislocation rate and, the excellent control of the leg length discrepancy [6]. Minimally invasive total hip replacement via the anterolateral approach in the supine position has yielded excellent clinical results [6, 7].

The conjoined external rotators tendon (CERT; conjoined tendons of the gemellus superior, obturator internus and, gemellus inferior) have been proposed as the key active stabilizers of the hip, along with the internally rotating gluteus minimus, are often described as the “rotator cuff” of the hip [8]. The CERT can help the external rotation of the hip and the stabilization of the hip joint [9] and is important to prevent posterior dislocation of the hip after THA [10, 11]. Although the anterior or anterolateral minimally invasive technique is reported as muscle-sparing THA, the surgeons sometimes need additional soft tissue release for canal preparation of the femur, such as the capsular ligament or the insertion of muscles around the hip including the CERT. These releases enable good canal preparation, insertion of broaches, and trials.

Currently, the clinical effects of CERT release in DAA THA are being investigated, only a few studies have described the effects of CERT release in DAA THA [12]. Consequently, the effects of CERT release in ABMS THA remain unresolved. The purpose of this study was to evaluate the clinical and radiographic results of CERT release in the anterolateral muscle-sparing THA approach.

## Materials and Methods

This was a single-center, retrospective data collection study, approved by our hospital’s institutional review board. The study considered for inclusion 92 hips from 92 patients who underwent unilateral primary THA with ABMS in the supine position at our institution between July 2017 and August 2018. The indication of THA with ABMS in this study was 1) no history of previous surgery on the affected hip, 2) primary osteoarthritis of the hip and osteonecrosis, and 3) secondary osteoarthritis of the hip with Crowe classification from 1 to 3. All hips were examined clinically and radiographically at 6 months and 1 year after surgery. Of these, three hips with opposite THA, two hips with total knee arthroplasty, and one hip with the tibial fracture within 12 months, and one hip with the onset of cerebral infarction on the perioperative period were excluded from the analysis. Finally, in total, 85 hips from 85 patients were included in this study. The baseline demographic data are listed in Table 1, including the age, gender, body mass index (BMI), follow-up periods, and diagnosis.

**Table 1:** Patient demographic data

Number of patients	85
Age at the operation, mean ± SD (range)	66.4 ± 8.1 (46 - 87)
Gender Female:Male, no. of patients (%)	77(90.6) : 8(9.4)
BMI	24.7 ± 3.2 (15.9 – 34.2)
follow-up period, mean ± SD (months) (range)	34.5 ± 3.7 (28 – 42)
Diagnosis, Stage (no. of hips)	
Osteoarthritis of the hip	84
Traumatic osteonecrosis	1

Data represent mean ± standard deviation

### Surgical technique

All surgeries were performed via the anterolateral muscle-sparing approach in the supine position (ABMS-THA) [6]. Both legs were draped, and the length of the skin incision ranged between 7 and 10 cm. The muscular plane between the gluteus medius and tensor fascia latae was developed. The anterior capsule was incised, including a vertical band of the iliofemoral ligament that was left about 10 mm from the medial border of the anterior capsule. The femoral neck was cut without a hip dislocation, and the femoral head was removed. The cup was inserted at a 40° inclination based on the interconnection line of the bilateral anterior–superior iliac spine and alignment rod for the inclination of cup positioner, and at an anteversion angle of 15° with reference to the operation table and cup positioner.

The preparation for stem insertion was performed. The capsular which remained, including a horizontal band of the iliofemoral ligament at the anterior part of the medial surface of the greater trochanter, was carefully resected. The CERT was confirmed and was retained as much as possible to provide stability. Regarding the CERT release, it was determined by the difficulty associated with the exposure of the canal of the femur during surgery. The CERT was released at the attachment site from the anterior to the posterior direction to achieve alignment [13]. If an additional elevation or lateralization of the femur was required, the external obturator muscle and posterior capsule attachments to the posterior part of the femoral neck were released as needed.

The head size, length, and offset were determined based on pre-operative templating, and minor adjustments were made intraoperatively. On the acetabular side, G7 cup and E1 acetabular flat liner (Zimmer Biomet, Warsaw, IN, USA) were used. On the femoral side the implant choice was made depending on the femoral shape or anteversion, Taperloc/Microplasty in 78 hips, Wagner cone in four hips, CL Spotorno in two hips (Zimmer Biomet, Warsaw, IN, USA), and C-stem AMT in one hip (DePuy Synthes, Warsaw, IN, USA). In the post-operative rehabilitation, patients were allowed immediate full-weight-bearing with a walker or crutches.

### Evaluation

Evaluation of the health status included the assessment of BMI. The duration of surgery, amount of blood loss during the operation, and post-operative complications were recorded.

Clinical assessments were performed using the Japanese Orthopaedic Association (JOA) hip scores [14] as the clinician-reported outcome measures (CROMs) pre-operatively, and at 6 and 12 months post-operatively. Additionally, patient-reported outcome measures (PROMs) were evaluated with the Japanese Orthopaedic Association Hip-disease Evaluation Questionnaire (JHEQ) [15, 16] and Forgotten Joint Score (FJS) [17]. The JHEQ consisted of pain, movement, and mental subscales, which were assessed pre-operatively, at 6 and 12 months post-operatively. The FJS was useful in Japan [18], and evaluated at 6 and 12 months post-operatively. The individual patient health status was monitored based on the short-form 36 questionnaires (SF-36 mental and physical) which included physical (Physical component summary: PCS) and mental sections (Mental component summary: MCS) pre-operatively and at 12 months postoperatively [19–21]. The questionnaires were completed by the patients themselves.

Conventional anteroposterior radiographs of the pelvis, including both hips and cross-table lateral views of the hip, were obtained to check the implant fixation. The pre-operative and post-operative leg length discrepancy (absolute value), cup inclination, stem orientation (valgus or varus angles), and subsidence of stem were assessed with anteroposterior radiographs. The cup anteversion and stem sagittal alignment (flexion or extension angles) were evaluated based on lateral views.

### Statistical analysis

Statistical analyses were performed using the EZR (Saitama Medical Centre, Jichi Medical University, Saitama, Japan), which is a graphical user interface for R (The R Foundation for Statistical Computing, Vienna, Austria) [22]. Specifically, it is a modified version of the R commander designed to add statistical functions and is frequently used in biostatistics. Wilcoxon signed-rank tests were used to compare the pre-operative and post-operative clinical and radiographic scores. The comparisons of continuous variables were compared with the Mann–Whitney U test. Fisher’s exact tests were used to compare demographic data between the groups. The level of significance was set at p = 0.05.

## Results

Among all the included hips, 37 hips (43.5%) retained a CERT (Non-released group) and 48 hips (56.5%) included a CERT release (Released group). The mean ages and BMI were not significantly different (Table 2). The duration of surgery was significantly longer and the amount of blood loss was more extensive in the released group (Table 2). The four hips needed an additional release of the external obturator muscle attachment.

**Table 2:** JOA and JHEQ scores preoperatively, and at 6 and 12 months postoperatively

	Non Released	Released	p value
Number	37	48	
Age	66.2 ± 7.9	67.4 ± 8.7	0.35
BMI	24.5 ± 3.2	24.8 ± 3.5	0.59
Duration of surgery	80.7 ± 16.1	93.0 **±** 23.0	0.00*
Amount of blood loss	304.8 **±** 150.4	369.8 ± 144.4	0.01*
Osteoarthritis grade			
Tonnis grade 1	4	3	0.75
Tonnis grade 2	4	6	
Tonnis grade 3	29	39	
The number of additional release			
the external obturator muscle attachment	0	3	
posterior capsule attachment to the posterior	0	5	
Stem selection			
Taperloc/ Microplasty	36	42	
Wagner cone	0	4	
CL Spotorno	0	2	
C-stem AMT	1	0	
Complication			
dislocation	0	0	
infection	0	0	
great trochanter fracture	1	1	
subsidence of stem, more than 3mm	2	1	

Data represent mean ± standard deviation

*P<0.05

Mann Whitney U test was performed to compare scores between the groups

Fisher’s exact test to compare numbers in OA grade and stem selection between the groups

The JOA hip score, all JHEQ scores, and PCS score of the SF-36 were significantly improved without the MCS score (Table 3). There were no significant differences in the JOA hip score, JHEQ, FJF-12, and SF-36 between the non-released group and released groups (Tables 4, 5).

**Table 3:** JOA, JHEQ and Summary scores of SF-36 before operation, and at 6 and 12 months postoperatively

	pre operation	at 6 months	p value	at 12 months	p value
JOA score	44.8 ± 12.7	95.2 ± 5.8	0.000*	95.7 ± 7.4	0.00*
JHEQ score					
total (84)	20.7 ± 10.3	65.8 ± 12.0	0.000*	70.2 ± 10.7	0.00*
pain (28)	6.4 ± 4.0	25.4 ± 3.5	0.000*	26.0 ± 2.8	0.00*
movement (28)	4.0 ± 3.5	18.7 ± 6.0	0.000*	20.8 ± 5.7	0.00*
mental (28)	10.4 ± 5.6	22.2 ± 5.5	0.000*	23.8 ± 4.5	0.00*
dissatisfaction scale (100)	87.3 ± 11.8	8.7 ± 12.1	0.000*	8.9 ± 14.8	0.00*
SF-36 PCS	22.3 ± 10.0	39.3 ± 11.7	0.000*	41.7 ± 11.3	0.00*
SF-36 MCS	56.0 ± 9.3	57.9 ± 8.1	0.094	56.8 ± 9.2	0.74

Data represent mean ± standard deviation

*P<0.05

Willcoxon single rank test was performed to compare scores

between pre operration and 6 and 12 months after surgery

**Table 4:** JOA and JHEQ scores between two groups

	Non Released	Released	p value
JOA score			
pre operation	44.3 ± 14.3	45.2 ± 11.4	0.6
at 6 months	96.0 ± 5.6	94.6 ± 6.0	0.32
at 12 months	94.9 ± 9.7	96.3 ± 4.9	0.74
JHEQ score			
pre operation			
total (84)	21.2 ± 10.1	20.3 ± 10.6	0.8
pain (28)	6.5 ± 4.4	6.3 ± 3.8	0.97
movement (28)	3.7 ± 3.3	4.2 ± 3.6	0.53
mental (28)	11.0 ± 5.3	9.9 ± 5.9	0.51
dissatisfaction scale (100) at 6 months	86.1 ± 12.3	88.2 ± 11.4	0.35
total (84)	68.8 ± 11.8	64.9 ± 11.8	0.12
pain (28)	25.9 ± 3.7	25.0 ± 3.4	0.23
movement (28)	19.9 ± 6.0	17.9 ± 5.9	0.1
mental (28)	23.2 ± 5.5	22.8 ± 5.5	0.42
dissatisfaction scale (100) at 12 months	9.3 ± 13.0	8.52 ± 11.6	0.91
total (84)	70.7 ± 11.9	69.8 ± 9.7	0.4
pain (28)	26.5 ± 2.6	25.6 ± 2.8	0.12
movement (28)	20.8 ± 6.7	20.9 ± 4.9	0.48
mental (28)	23.8 ± 4.7	23.8 ± 4.4	0.72
dissatisfaction scale (100)	7.4 ± 15.3	10.1 ± 14.4	0.14

Data represent mean ±standard deviation

*P<0.05

Mann Whitney U test was performed to compare scores between groups

**Table 5:** Forgotten joint score and Summary score of SF-36 between two groups

	Non Released	Released	p value
Forgotten joint score			
at 6 months	66.9 ± 18.5	58.5 ± 19.8	0.06
at 12 months	74.1 ± 15.2	72.5 ± 13.8	0.64
SF-36 PCS			
pre operation	22.7 ± 11.5	22.1 ± 8.7	0.83
6 months	39.4 ± 12.4	39.2 ± 11.3	0.94
12 months	42.7 ± 11.2	41.0 ± 11.5	0.56
SF-36 MCS			
pre operation	55.7 ± 8.2	56.2 ± 10.1	0.86
6 months	58.6 ± 8.0	57.4 ± 8.2	0.31
12 months	56.5 ± 9.6	57.0 ± 9.0	0.87

Data represent mean ± standard deviation

*P<0.05

Mann Whitney U test was performed to compare scores between groups

There were significant differences in the stem flexion angle between the groups. The pre-operative and post-operative leg length discrepancies, extension lengths, cup inclinations, anteversions, and stem varus angles were not significant (Table 6).

**Table 6:** Radiographic parameters

	Non Released	Released	p value
preoperative leg length discrepancy	-7.9 ± 8.9	-9.9 ± 9.5	0.71
postoperative leg length discrepancy	0.2 ± 6.8	-0.9 ± 9.0	0.76
Leg extension length	7.2 ± 6.2	8.5 ± 8.1	0.36
cup inclination	42.5 ± 5.5	44.7 ± 4.5	0.06
cup anteversion	18.2 ± 5.4	20.0 ± 6.0	0.46
stem varus angle	1.9 ± 5.4	1.2 ± 2.5	0.78
stem flexion angle	7.2 ± 3.5	4.2 ± 3.8	0.04*

Data represent mean ± standard deviation

*P<0.05

Mann Whitney U test was performed to compare scores between groups

## Discussion

The clinical results of this study had excellent post-operative outcomes for THA, given that the hip clinical scores were significantly improved (with the exception of the mental section of SF-36), regardless of the CERT release. Furthermore, the leg length discrepancy was improved. The ABMS for THA with preserved CERT was less invasive, the duration of surgery was significantly longer, and the amount of blood loss was more extensive in the released group.

Although the deep external rotators have been proposed as key active stabilizers of the hip described as the ‘rotator cuff’ of the hip [8], the CERT release during the DAA in THA has no impact on gait or patient-reported outcomes within the 12-week period post-surgery [23]. Yao et al. investigated the outcome of DAA for THA and showed that the CERT release was not predictive of the length of stay, one year inpatient pain medication requirements, or outcome scores [12]. These results were consistent with the results of the present study, wherein 56.5% of the hips included a conjoint tendon release irrespective of whether the CERT released or not in anterolateral muscle-sparing THA, and had limited effects on patient outcomes at six months and one-year based on the use of CROMs and PROMs. Specifically, FJS was considered as the most sensitive score and the ultimate goal in joint arthroplasty [17] and tended to show greater dispersion and more marked differences between patients [18].

The importance of preservation of CERT for post-operative hip stability has been described in the case of the posterior approach [24], to prevent posterior dislocation of the hip after THA [10, 11]. The data of the present study showed no dislocation, irrespective of whether the attachment of CERT (released or not) affected the post-operative stability in ABMS for THA. Conversely, if the CERT was released in the non-released group cases, joint instability may be increased.

A cadaveric study has shown considerable variation in the attachments of CERT [13]. The CERT insertion is mostly attached to the anterior part of the femur among the short external rotation muscles [25]. The damage of the obturator internal muscle depended upon the injury at CERT insertions [26] and induced a decrease of the muscle volume [9]. In the surgical steps of the present study, the CERT was released at the attachment site from the anterior to the posterior directions in the case of inadequate canal preparations. The CERT was related to the adjustment of the range of motion and the joint stability, the present study showed the limited effect on patient-reported outcomes, including pain and daily-life activities.

The radiographic evaluation showed that stem sagittal alignment was more flexion in the non-released group, while no significant differences existed in the stem valgus/varus alignment. The stem tended to be inserted from the anterior part of the osteotomy site of the femur if the canal preparation was inadequate. Yoshitani et al. reported that there were no significant differences in clinical or radiographic outcomes between flexion and neutral alignment of the tapered-wedge stem at an average follow-up period of 4.7 years [27]. The difference in the sagittal alignment of our data had a limited effect on the clinical course.

Our study has certain limitations. The first limitation is that this study was a nonrandomized control study. The CERT release depended on the cases, and hips with contracture may have tended to release the capsular band and CERT. The second limitation was stem selection. The choice of the stem was according to the shape of the femur, anteversion, and offset; the differences in the shape of the stem shoulder or stem length affected the capsular band and CERT release, and femur canal preparation. The third limitation pertains to the fact that our study did not include shorter-term outcomes. We may detect short-term clinical differences if shorter-term outcomes were examined.

## Conclusions

The CERT release in anterolateral muscle-sparing total hip arthroplasty has a limited effect on postoperative clinical outcomes. Although the CERT release improves femur exposure, the CERT has limited effects on patient outcomes at 6 months and one year post-surgery. The CERT release was more invasive than the preserved CERT, and the thresholds levels may be low. Surgeons should try to leave the CERT for patients with a wide range of motion as much as possible, and release the CERT for inadequate femur canal preparation.

## Data Availability

The datasets used and/or analyzed during the current study are available from the corresponding author on reasonable request.

## Abbreviations

THA: total hip arthroplasty
ABMS: anterior-based muscle-sparing
DAA: direct anterior approach
CERT: conjoined external rotators tendon
BMI: body mass index
JOA: Japanese Orthopaedic Association
CROMs: clinician reported outcome measures
PROMs: patient-reported outcome measures
JHEQ: Japanese Orthopaedic Association Hip-disease Evaluation Questionnaire
FJF: forgotten joint score
SF-36: 36-Item. Short-Form Health Survey
PCS: Physical component summary
MCS: Mental component summary
